# The impact of various forms of exercise-induced fatigue on the short-passing performance of sub-elite female football players

**DOI:** 10.3389/fspor.2026.1734883

**Published:** 2026-04-17

**Authors:** M. Sparks, A. Strauss, C. Pienaar

**Affiliations:** 1 Physical Activity, Sport and Recreation (PhASRec), Faculty of Health Sciences, North-West University, Potchefstroom, South Africa; 2 Department of Sport, Rehabilitation & Dental Science, Tshwane University of Technology, Pretoria, South Africa; 3 Department of Sport Studies, Faculty of Applied Sciences, Durban University of Technology, Durban, South Africa

**Keywords:** endurance, female football, maximal sprint, passing performance, soccer fatigue, technical skills

## Abstract

**Introduction:**

The purpose of this study was to describe physical and short-passing performance of sub-elite female soccer players and to determine the effects of fatigue induced by different exercise protocols on passing performance.

**Methods:**

Forty-eight South African female football players participated in the study. Players completed the Loughborough Soccer Passing Test (LSPT) before and directly following the execution of two fatigue-inducing protocols: a repeated sprint ability (RSA) test and the Yo-Yo Intermittent Recovery Level 1 (Yo-Yo IR1) test. Peak heart rate and blood lactate concentration were obtained following the fatiguing exercises.

**Results:**

A decline in the LSPT performance was found in terms of passing, penalty and total time following both fatiguing exercises. A significant (*p* < 0.001) increase in penalty time (32.6%) and total performance time (10.1%) was recorded following the Yo-Yo IR1. Penalty time (20.4%) and total performance time (8.5%) also increased following the RSA test. Moderate to high correlations were found between Yo-Yo IR1(r = −0.47 – −0.51) and RSA (r = −0.40 – −0.48) results with baseline LSPT performance. Percentage decline in LSPT performance was not significantly associated with Yo-Yo IR1 and RSA performance.

**Discussion:**

Physical fatigue has a detrimental effect on short-passing ability, with aerobic fatigue influencing passing accuracy more than anaerobic fatigue, resulting in a larger decline in short-passing performance. While higher fitness levels were associated with superior baseline short-passing performance, they did not protect players from the relative deterioration of skills once fatigued.

## 1 Introduction

Over the last decade, there has been a significant increase in the quantity of research done on women's football (or soccer), which reflects the popularization and professionalization of women's football (1). Consequently, the profiles of female players and their development towards becoming elite performers are of decisive interest in the football milieu (2). During matches, elite female players typically cover between 8 and 11 km, reflecting the high physical demands of the game (3–6). To meet these demands, a well-developed aerobic capacity is essential (7). However, football is not solely an endurance-based sport; players are also required to execute numerous short-duration sprints (<10 s) interspersed with brief recovery periods (<30 s) or low- to moderate-intensity activity (8). This quality, known as repeated-sprint ability (RSA), is widely recognized as a critical component of high-intensity intermittent sports such as football (9). Importantly, these physical demands occur alongside the continual need to execute technical skills, meaning that passing, dribbling, and shooting must often be performed under conditions of physical strain (10). As a result, understanding how fatigue influences short-passing execution is of particular relevance, since most actions in match play are carried out while players are experiencing some degree of fatigue.

Even though it is evident that match related fatigue results in a decline in physical performance, less research has been dedicated to the effect of fatigue on the quality of short-passing ability (11, 12). The deterioration of short-passing skills may be related to fatigue accumulated throughout a match and to the acute fatigue that is secondary to high-intensity phases of short duration (11). Limited experimental research is available in this area due to the difficulty in replicating the complex nature of football skills within a laboratory context (13). However, the Loughborough Soccer Passing Test (LSPT) has previously been used to assess skill performance at regular intervals during exercise (14), which can be utilized for examining the effect of fatigue on skill performance (13). The LSPT is the first football skill test to be validated for female players and is recommended to evaluate the football skill performance of female players in research settings (15).

The impact of fatigue on technical skill performance has been explored in male football players (11, 16–18) but needs further examination within female soccer. Due to the physiological demands being vastly different between male and female matches, the best training practices of males might be inaccurate when applied to the female football environment (19). A recent study investigated the effect of match simulated fatigue on the passing performance of elite and sub-elite female football players and found that their elite players were able to maintain their passing performance longer than sub-elite players. They furthermore indicated a need for more research on this topic (20). Despite the popularity of women's football, skill-related research in female football is underrepresented in Africa (21). Therefore, we aim to describe the physical and technical skill characteristics of sub-elite female football players and to determine the effect of exercise induced fatigue on the performance of short-passing skill performance results. We hypothesised that both exercise induced fatigue protocols would negatively affect short-passing performance in sub-elite female football players.

## 2 Material and methods

### 2.1 Participants

Forty-eight (*n* = 48) South African sub-elite female soccer players (age: 22.0 ± 2.7 years; height: 158.9 ± 5.8 cm; body mass: 55.5 ± 8.1 kg) participated in the study. Mckay et al. (22) defines Sub-elite/ highly trained/ National level participants as team-sport athletes competing in national and/ state leagues/tournaments. The final sample of 48 participants therefore provided approximately 80% power to detect the observed effect. All players were football players in local tournaments with an average of 7.5 years of playing experience. The players were assessed during the competitive season. Prior to the start of the study, the players provided written informed consent, and players were excluded if they were injured.

### 2.2 Design

The study made use of a quasi-experimental, one-group pretest-posttest design. A demographic questionnaire and a physical performance datasheet were used to gather information. The study was approved by the Health Research Ethics Committee (NWU-00055-15-S1) of the university where the study was conducted. Research was conducted according to the Declaration of Helsinki.

### 2.3 Intermittent high-intensity endurance protocol: Yo-Yo IR1 test

The Yo-Yo IR1, as described previously (23), was used to evaluate the intermittent high-intensity endurance capacity of each player. This test requires a strong aerobic contribution as well as a substantial glycolytic contribution. The maximal oxygen consumption ($\overset{∙}{V}O_{2\max}$) of each player was determined through a formula previously used for estimating $\overset{∙}{V}O_{2\max}$ from the Yo Yo IR1 total distance covered (24). A significant correlation (*p* < 0.05) was found between the Yo-Yo IR1 distance covered and $\overset{∙}{V}O_{2\max}$ (24), indicating that $\overset{∙}{V}O_{2\max}$ can be estimated from the Yo-Yo IR1 results (24).

### 2.4 Repeated high-intensity interval protocol: RSA test

The RSA test was used according to a previously described method by Boddington et al. (25). The 5-meter Multistage Shuttle run Test (MST) was structured with six cones spaced five meters apart in a straight line, covering a total distance of 25 meters. Players started behind the first cone and sprinted five meters to the second cone before returning to the start. They then ran ten meters to the third cone and back, continuing this pattern with increasing distances for 30 s per bout. Each sprint sequence was followed by a 35-second rest period, with the test repeated six times in total. The players were instructed not to pace themselves but to perform with maximal effort throughout the test. The distance covered by each player was approximated to the nearest 2.5 m during each 30-second shuttle, and this was used to determine the average total distance covered.

### 2.5 Heart rate and blood lactate monitoring

The players were fitted with a Polar Heart Rate Transmitter Belt (Polar Electro, Kempele, Finland) before performing both the Yo-Yo IR1 and the RSA tests to measure heart rate (HR) for each five second period while completing the fitness tests. Immediately after completion of the Yo-Yo IR1 and the RSA tests, a blood sample was collected from the fingertip of the left hand. A portable analyzer (Lactate Pro, Arkray, Japan) was used to measure blood lactate concentration in mmol/L and was calibrated before each blood sampling and used according to the manufacturer guidelines. The player's finger was cleaned with an alcohol swab before each measurement. The blood samples were only used to confirm that each player was fatigued following the fitness tests before commencement of the technical skills test.

### 2.6 Loughborough soccer passing test (LSPT)

**Figure 1:**
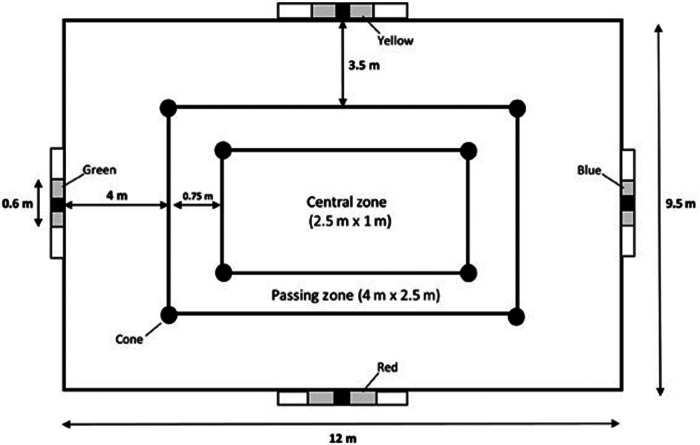
Schematic representation of the LSPT.

The LSPT was conducted to assess the multi-faceted aspects of football skills, including passing, dribbling, control and decision-making. Prior to the start of the study, the players were sufficiently familiarised with the LSPT protocol during a practice session. Figure 1 illustrates the layout of the LSPT. Participants completed 16 passes (8 long, 8 short) within a 9.5 m × 12 m grid, following a cone circuit. Four wooden benches (250 cm × 30 cm) served as targets, each with a coloured target area (60 cm × 30 cm) and a central black accuracy strip (10 cm × 30 cm). Participants started in the centre of the grid and moved into the designated passing zone before each pass. Passes were called out by an assessor in a predetermined but randomised colour order immediately before execution. An additional assessors recorded penalties. The aim was to complete all passes as quickly and accurately as possible. Final score was total time plus penalties: +5 s for missing the bench or wrong target; +3 s for missing the coloured area or handling the ball; +2 s for passing outside the designated zone; and +1 s for each second exceeding 43 s. One second was deducted for each strike of the black accuracy strip. The execution of the test and the awarded penalty time were in accordance with a previous study (15).

### 2.7 Procedure

All participants completed standardised familiarisation sessions prior to data collection to reduce potential learning effects. To further reduce any bias or fatigue effects testing was conducted over two consecutive days. Prior to testing, the players engaged in a general warm-up of 15 min consisting of low-intensity aerobic activity followed by static and dynamic stretching. On the first day, each player conducted the LSPT followed by the RSA test. Immediately following the RSA test, blood sampling was carried out. Then without any rest, the player completed the LSPT. On the second day, each player conducted the LSPT followed by the Yo Yo IR1 test. Immediately following the Yo-Yo IR1 test, blood sampling was carried out. Again, without any rest, the player then completed the LSPT. After completion of each fitness test, the player's maximal HR and rating of perceived exertion (RPE) were recorded. The Borg CR10 scale modified by Foster et al. (26) was used as a guideline to record the players’ perceived exertion during following each test (27). The test was conducted on a grass soccer field with players wearing their soccer boots. Players were instructed not to engage in strenuous exercise for at least 48 h before testing.

### 2.8 Statistical analysis

The Statistical Package for the Social Sciences (IBM SPSS Statistics 27) was used for statistical analysis. Data is presented as means ± standard deviation (SD). Descriptive statistics were calculated and used to describe the aerobic and anaerobic fitness characteristics of the players. Short-passing skill scores pre-and post the fitness tests were compared with a paired samples t-test after normality was confirmed (Shapiro–Wilk test). To determine practical significance, effect sizes (Cohen's D) were determined together with 95% confidence intervals. The magnitude of the effect size (ES) was described as follows: trivial (<0.2), small (0.2–0.59), moderate (0.6–1.19), large (1.2–1.99) or very large (>2.0) (28). Lastly, Spearman's Rank Correlation Coefficient was used to determine the relationship between the technical skill scores and the aerobic and anaerobic test results. The magnitude of correlation coefficients was considered as small (0.1 ≤ r < 0.3), moderate (0.3 ≤ r < 0.5), high (0.5 ≤ r < 0.7), very high (0.7 ≤ r < 0.9) and almost perfect (r ≥ 0.9) (28). The level of statistical significance was set at *p* < 0.05 for all tests. Five players did not attend the second day of testing, therefore did not complete the Yo-Yo IR1 test and did not form part of the analysis. Importantly, all participants who attended a given testing day completed both the pre- and post-test measurements for that session; thus, no within-session data was missing. A *post hoc* power analysis (one-tailed, *α* = 0.05) for the paired-samples *t*-test (dz = 0.41, *n* = 48) indicated that the study achieved a statistical power of 0.87 (87.1%) to detect the observed effect based on G*Power (v.3.1.9.7) analysis.

## 3 Results

Descriptive statistics for the Yo-Yo IR1 and RSA tests are presented in Table 1. Peak HR was slightly higher following the Yo-Yo IR1 than after the RSA test. Blood lactate measured after the RSA test was higher than blood lactate measured after the Yo-Yo IR1. The RPE scores following the Yo-Yo IR1 and the RSA test were similar. At volitional exhaustion during progressive incremental exercise, blood lactate concentrations typically reach 8–10 mmol/L, reflecting substantial metabolic stress (29). The post-exercise lactate values in our study were higher than this range, indicating a high level of physiological strain. Additionally, participants achieved ≥90% of their age-predicted maximal heart rate (208−[0.7 × age), consistent with near-maximal cardiovascular effort. Together, these responses confirm that participants experienced sufficient physiological stress to induce fatigue.

**Table 1:** Descriptive statistics (mean ± SD) of the Yo-Yo Intermittent Recovery Test level 1 (Yo-Yo IR1) and the Repeated Sprint Ability Test (RSA).

Variables	Yo-Yo IR1 (95% CI) (*n* = 43)	RSA (95% CI) (*n* = 48)
Total distance (m)	560.9 ± 212.8 (495.4–626.4)	614.3 ± 41.9 (602.1–626.4)
Peak HR (bpm)	190.1 ± 8.0 (187.6–192.5)	186.2 ± 8.9 (183.7–188.8)
Peak blood lactate (mmol/L)	10.9 ± 3.5 (9.8–12.0)	16.4 ± 4.3 (15.1–17.6)
RPE	7.1 ± 1.9 (6.5–7.7)	7.2 ± 2.0 (6.6–7.8)
$\overset{∙}{V}O_{2\max}$ (mL/kg/min)	41.1 ± 1.8 (40.6–41.7)	
Yo-Yo IR1 level	14.1 ± 0.9 (13.8–14.4)	

SD, standard deviation.

**Figure 2:**
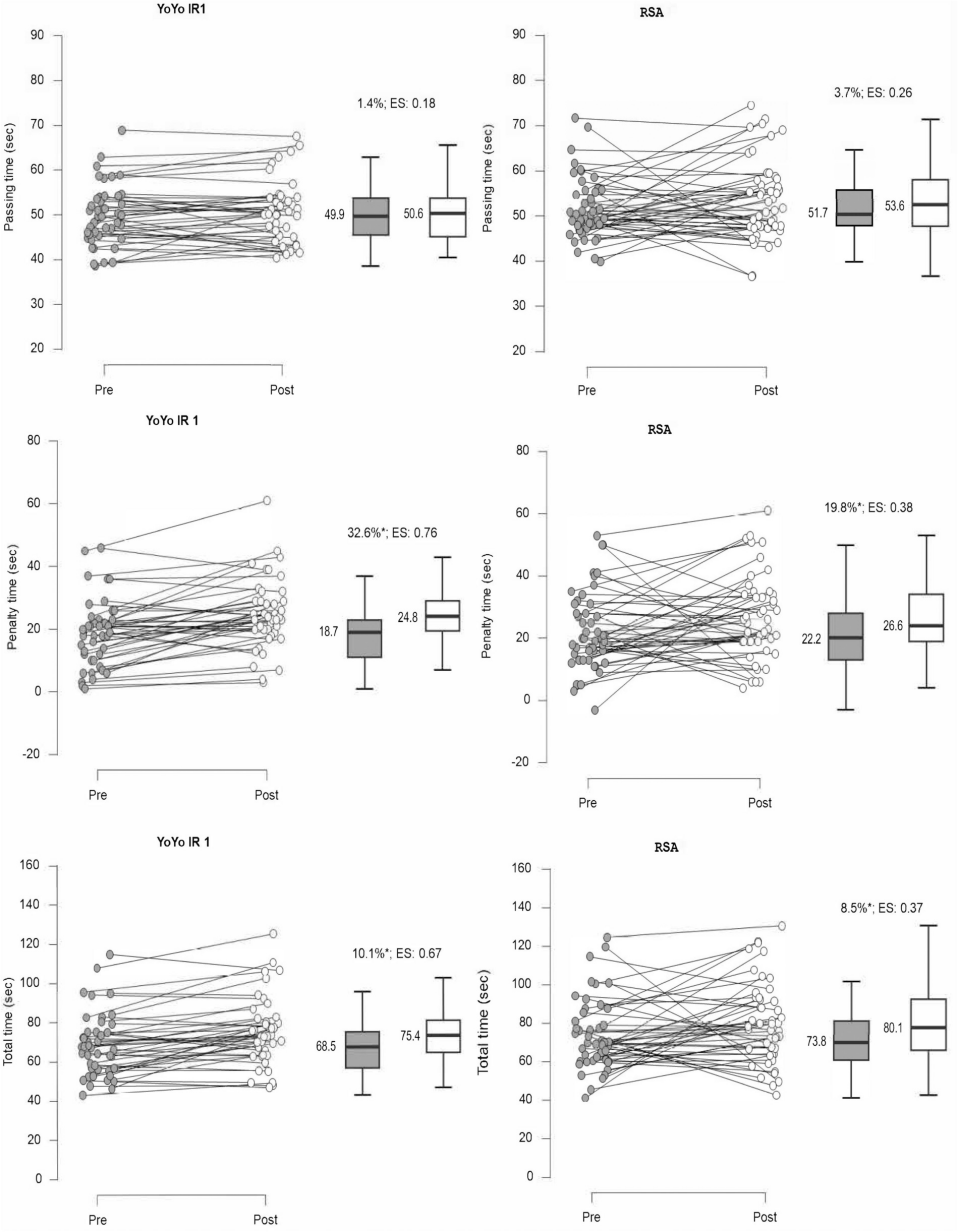
Graphical presentation of LSPT scores for each participant preceding (pre-test) and following (post-test) the Yo-Yo IR1 (*n* = 43) and RSA test (*n* = 48). *p < 0.05.

**Figure 3:**
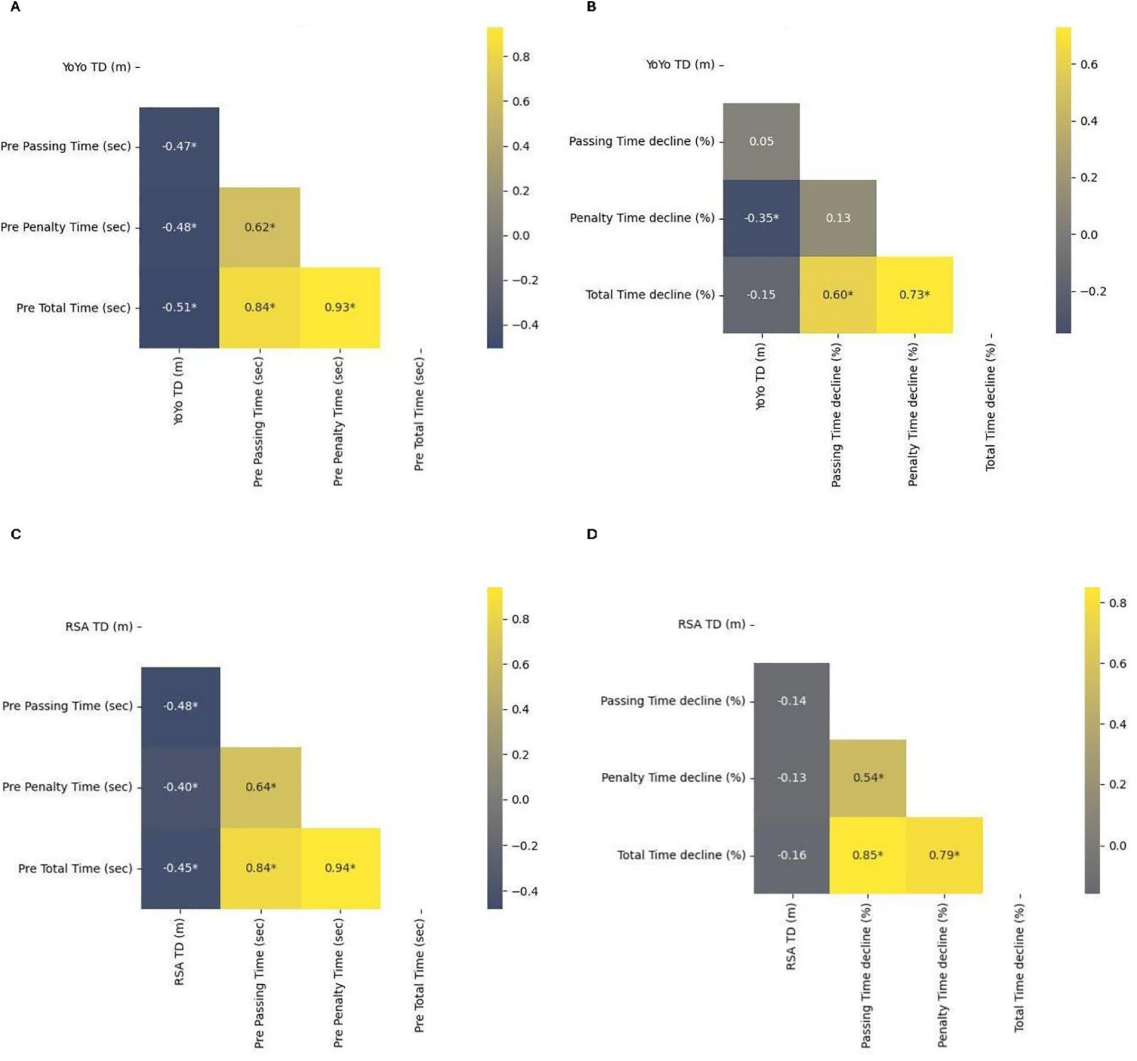
Heatmap of correlations between Yo-Yo IR1 results and LSPT performance **(A)**, Yo-Yo IR1 results and relative decline in LSPT performance **(B)**, RSA results and LSPT performance **(C)**, RSA results and relative decline in LSPT performance **(D)** *p < 0.05.

A summary of the LSPT performance scores and a graphical presentation of each data point is presented in Figure 2. The performance score comprised three variables: time taken to complete the LSPT; penalty time accrued; and total performance time. The performance in the LSPT decreased during the post tests that followed the fatiguing exercises. The LSPT passing time increased following both the Yo-Yo IR1 and RSA tests. In particular, penalty time increased after the Yo-Yo IR1 significantly with a moderate effect size (ES = 0.76; 32.6%), which also led to significantly high effect size increase for total time (ES = 0.67; 10.1%). There was also a small but significant increase in the penalty time (ES = 0.38; 19.8%) and total time (ES = 0.37; 8.5%) after the RSA test. In Figures 3A, B the correlation matrix for the Yo-Yo IR1 and LSPT performance are presented. The results revealed strong evidence that Yo-Yo IR1 performance is negatively associated with baseline LSPT performance (r = −0.47 to −0.51; *p* < 0.05), with a moderate negative relationship presented for Yo-Yo IR1 performance and relative penalty time decline (r = −0.35; *p* < 0.05) in the LSPT after the Yo-Yo IR1. Regarding the RSA results (Figures 3C, D), moderate to high evidence was found that RSA performance was negatively associated with baseline LSPT performance (r = −0.40 to −0.48; *p* < 0.05). No evidence was found that RSA performance was associated with LSPT performance decline after the RSA was performed.

## 4 Discussion

The purpose of this study was to describe the physical and short-passing skill characteristics of sub-elite female soccer players and to determine the effects of fatigue induced by different exercise protocols on the performance of short-passing skills. The main findings of this study were i) both intermittent high-intensity endurance (induced by the Yo-Yo IR1) and intermittent maximal sprint fatigue (induced by the RSA) resulted in significant declines in short-passing performance, ii) the decline was greater following Yo-Yo IR1, and iii) Yo-Yo IR1 and RSA fitness were significantly related to pre-fatigue short-passing performance, but not with the relative percentage decline after the fatiguing test. Overall, the results suggest that while fitness is related to an advantage in absolute short-passing performance levels, it does not prevent the relative deterioration of short-passing skills under fatigue.

Short-passing skill performance decreased significantly in both experimental conditions. This is consistent with past studies, which found that players' performance declined after high-intensity interval training (20, 30). Our findings reflect this decline in performance, with passing accuracy and completion speed both declining as players fatigued, mirroring the demands of match play, where many crucial technical movements occur under fatigue (3, 6). Previous research suggested that the decline in short-passing performance was due to a decrease in muscle function capacity which in turn leads to a decrease in stability and accuracy in passing (31). The increase in penalty times in our study suggests that players attempted to maintain execution speed at the expense of accuracy, reflecting a “speed–accuracy trade-off” that has been consistently observed in soccer-specific skill testing (10). These findings highlight the importance of examining skills under realistic, fatigued conditions rather than in isolation.

A novel contribution of this study is the direct comparison of intermittent high-intensity endurance and intermittent maximal sprint fatigue. Although both protocols led to skill deterioration, the Yo-Yo IR1 produced a greater decline in performance than the RSA. Added penalty time makes up most of the difference between the Yo-Yo IR1 and the RSA test, suggesting that intermittent high-intensity endurance fatigue hinders performance more than intermittent maximal sprint fatigue. Importantly, the larger technical deterioration after the Yo-Yo IR1 test emphasizes the greater challenge of sustaining skill execution under progressive, endurance-related fatigue compared to shorter, intermittent fatigue. This aligns with match-play realities, where prolonged periods of high-intensity running are often associated with reduced passing accuracy and decision-making efficiency. In contrast to the progressive fatigue build up during the Yo-Yo IR1 as a result of the longer time duration involved in completing the test (approximately 14 min), sufficient recovery was promoted during the shorter RSA test (approximately 6 min incl. rest). Thus, while intermittent maximal sprint fatigue is acute and intense, intermittent high-intensity endurance fatigue appears to place a heavier cumulative burden on technical execution.

The lactate and RPE data provide additional context for interpreting the skill declines. Based on the interpretation of the RPE scale used, players experienced the fatiguing exercises as very difficult, and together with the high blood lactate levels, the tests were of sufficient intensity to exhaust the players before the LSPT. Interestingly, lactate concentrations were higher following the RSA (16.4 mmol/L) than the Yo-Yo IR1 (11.2 mmol/L), yet skill performance deteriorated more after the Yo-Yo IR1. In a study investigating small-sided games, regimes involving passive rest resulted in higher lactate responses in comparison to active rest (32). The active resting regime used in the Yo-Yo IR1 may, therefore, enhance blood lactate removal in comparison to the passive resting regime of the RSA test, which may explain the higher blood lactate concentration measured following the RSA test.

In addition to physiological factors, cognitive demands could have contributed to the observed results as well. Technical skill performance may be influenced by cognitive functions and concentration (11, 17, 33). Perceptual abilities (reaction time, decision making and anticipation) are important during competitive play and for the successful execution of soccer skills (16, 33). Although cognitive functioning and specifically concentration was not measured in this study, immediately following the fatiguing exercises, many players seemed unsteady and more focused on the fatigue than the passing task at hand. Mental weariness has a direct effect on performance with players mentally weary experiencing an impaired ability to concentrate, an increase in reaction times and an increased risk to make mistakes (31). Furthermore, several studies found that mental fatigue reduces passing accuracy and ball speed (34–36). A possible mechanism for this decline in performance is a decreased ability to anticipate ball movement and preparation in controlling it (34). This is supported by the increase in overall passing time and penalty time during this study.

The role of fitness and its relationship with technical skill execution was also investigated in this study. Both Yo-Yo IR1 and RSA scores were significantly associated with baseline short-passing skill performance, indicating that players with higher fitness also demonstrated superior technical ability. This suggests that fitness may underpin a player's capacity to consistently execute technical skills in fresh states. This agrees with a previous study where researchers did an aerobic fitness intervention and found an improvement in LSPT performance after the four-week intervention (37). Similarly, Zago et al. (38) found that after a high-intensity intervention, consisting of several maximum sprinting efforts, players improved their LSPT performances significantly. Even though there were significant relationships between fitness and short-passing performance in our study, the absence of significant correlations between fitness measures and percentage decline indicates that higher fitness does not necessarily buffer against the relative impact of fatigue. In other words, fitter players perform better in absolute short-passing skills terms but experience a similar proportional deterioration once fatigue sets in.

From an applied perspective, these findings highlight the necessity of preparing players to execute technical skills under fatigue. Training drills that combine physical conditioning with skill execution — such as small-sided games, high-intensity interval drills with integrated passing, or skill tasks performed post-running — may better replicate match demands and improve resilience of technical performance. Coaches should also recognize that fitter players are likely to sustain higher levels of technical skill performance across matches, though all players are susceptible to skill decline when fatigued. Although research on African female football players is necessitated, this study was limited to a relatively small sample of female players, restricting the generalizability of the findings. Additionally, environmental conditions were not formally recorded during testing. However, to encourage consistency in environmental exposure, all testing sessions were held at the same location for every team, during the same time of year, at comparable times of day, and on consecutive days. While both fatigue protocols were well-validated, female-specific RSA protocols remain underrepresented in the literature. Future research should examine how perceptual-cognitive skills (e.g., decision-making, visual scanning) interact with technical performance under fatigue, and whether targeted training can mitigate the detrimental effects observed here.

To conclude, this study demonstrated that both exercise protocols to induce fatigue negatively affect the short-passing performance of sub-elite female football players, with intermittent high-intensity endurance fatigue (Yo-Yo IR1) producing a greater decline than intermittent maximal sprint fatigue (RSA). While higher fitness levels were associated with superior baseline short-passing performance, they did not protect players from the relative deterioration of skills once they were fatigued. These findings highlight the importance of integrating conditioning and technical skill execution in training to better prepare players for the demands of match play. Moreover, the results contribute valuable insights into the limited body of knowledge on African female football players, underlining the importance of context-specific research for this underrepresented population.

## Data Availability

The raw data supporting the conclusions of this article will be made available by the authors, without undue reservation.
